# Clinical decision support system using hierarchical fuzzy diagnosis model for migraine and tension-type headache based on International Classification of Headache Disorders, 3rd edition

**DOI:** 10.3389/fneur.2024.1444197

**Published:** 2024-09-10

**Authors:** Ziming Yin, Heng Li, Xun Han, Ye Ran, Zhichen Wang, Zhao Dong

**Affiliations:** ^1^School of Health Science and Engineering, University of Shanghai for Science and Technology, Shanghai, China; ^2^Department of Neurology, International Headache Center, Chinese PLA General Hospital, Beijing, China; ^3^International Headache Center, Chinese PLA General Hospital, Beijing, China

**Keywords:** migraine, tension-type headache, fuzzy logic, artificial intelligence, ICHD-3, CDSS

## Abstract

**Objective:**

To determine whether the diagnostic ability of the newly designed hierarchical fuzzy diagnosis method is consistent with that of headache experts for probable migraine (PM) and probable tension-type headache (PTTH).

**Background:**

Clinical decision support systems (CDSS) are computer systems designed to help doctors to make clinician decisions by information technology, and have proven to be effective in improving headache diagnosis by making medical knowledge readily available to users in some studies. However, one serious drawback is that the CDSS lacks the ability to deal with some fuzzy boundaries of the headache features utilized in diagnostic criteria, which might be caused by patients’ recall bias and subjective bias.

**Methods:**

A hybrid mechanism of rule-based reasoning and hierarchical fuzzy diagnosis method based on International Classification of Headache Disorders, 3rd edition (ICHD-3) was designed and then validated by a retrospective study with 325 consecutive patients and a prospective study with 380 patients who were clinically diagnosed with migraine and TTH at the headache clinic of Chinese PLA General Hospital.

**Results:**

The results of the diagnostic test in the retrospective study indicated that the fuzzy-based CDSS can be used in the diagnosis of migraine without aura (MO) (sensitivity 97.71%, specificity 100%), TTH (sensitivity 98.57%, specificity 100%), PM (sensitivity 91.25%, specificity 98.75%) and PTTH (sensitivity 90.91%, specificity 99.63%). While in the prospective study, the diagnostic performances were MO (sensitivity 91.62%, specificity 96.52%), TTH (sensitivity 92.17%, specificity 95.47%), PM (sensitivity 85.48%, specificity 98.11%) and PTTH (sensitivity 87.50%, specificity 98.60%). Cohen’s kappa values for the consistency test were 0.984 ± 0.018 (MO), 0.991 ± 0.018 (TTH), 0.916 ± 0.051 (PM), 0.932 ± 0.059 (PTTH) in the retrospective study and 0.884 ± 0.047 (MO), 0.870 ± 0.055 (TTH), 0.853 ± 0.073 (PM), 0.827 ± 0.118 (PTTH) in the prospective study, which indicated good consistency with the fuzzy-based CDSS and the gold standard (*p* < 0.001).

**Conclusion:**

We developed a fuzzy-based CDSS performs much more similarly to expert diagnosis and performs better than the routine CDSS method in the diagnosis of migraine and TTH, and it could promote the application of artificial intelligence in the area of headache diagnosis.

## 1 Introduction

Migraine and tension-type headache (TTH) are the two most common primary headache disorders, demonstrating high prevalence and socioeconomic impacts (1, 2). However, the diagnostic accuracy is only 13.8% for migraine and 5.6% for TTH according to a previous population-based study in China (3), which might lead to excessive neuroimaging examination, delayed preventive treatment, and even medication overuse.

In our previous study (4), we developed a rule-based clinical decision support system (CDSS) based on the International Classification of Headache Disorders (ICHD) (5) and achieved relatively satisfactory diagnostic accuracy for most primary headache disorders. To address the diagnostic difficulty due to the overlap between primary headaches (6–8), we also used a case-based reasoning method and ultimately increased the diagnostic accuracy for probable migraine and probable tension-type headache (9). However, one obvious drawback is that these two CDSSs lack the ability to deal with some fuzzy headache features (i.e., the duration of attacks, headache intensity and number of attacks), which are often subject to recall bias and subjective bias. Regarding the duration of attacks and the number of attacks, it is known that patients often have difficulty recalling their precise headache features. During the clinical interview, the information concerning the frequency and temporal pattern of attacks and days with headache(s) that patients provide often includes rough and imprecise estimates, and this can interfere with the quantification of the real number of headache days per month (10). Regarding headache intensity, it has been reported that recall of headache is easily affected by subjective factors, such as the mood or stress at the time of pain perception, pain intensity or mood at the time of recall, peak pain intensity, pain intensity at the end of the period and variability of pain intensity. Therefore, there may be discrepancies between the recalled and actual headache intensity (11). The imprecise description of these three headache features due to recall bias and subjective bias means that experienced doctors must deal with the boundaries of headache features in a fuzzy and approximate way, rather than using a one-size-fits-all approach as in the diagnostic criteria in ICHD. The rule-based CDSS and case-based CDSS cannot use such an approach. Therefore, it is necessary to develop a new headache diagnostic model to address fuzzy features and fuzzy information.

Fuzzy logic is a kind of artificial intelligence technology that is useful in expressing the intrinsic uncertainty and unclear boundaries of the patient features utilized in auxiliary diagnosis, and it has been found to be useful in a number of disease diagnoses, such as unstable angina (12), osteoporosis (13), and breast cancer (14). Fuzzy logic can imitate the processing of fuzzy concept judgements and reasoning as an expert does. For a system with a fuzzy model, fuzzy sets and fuzzy rules can be applied to express the transitional boundary of qualitative knowledge and experience and to solve the problem that the conventional method is difficult to use. Specially, the fuzzy diagnostic modelling of ICHD-3 is a process of translating the text-based diagnostic criteria in ICHD to digital executable models for computers with the cooperation of headache experts and knowledge engineers. Hierarchical fuzzy logic is a method of hierarchical modelling that can decompose a complex process and execute it in a sequence from simple to complex. This method has been successfully applied in other field (15, 16). In this study, we aimed to optimize the diagnostic modelling method of migraine and TTH and to design a hierarchical fuzzy inference method that can be regarded as a supplement to rule-based reasoning and case-based reasoning in the headache CDSS. Furthermore, we clinically evaluated the validity and feasibility of this system in a headache clinic of Chinese PLA General Hospital by a retrospective study and a prospective study respectively. Besides, we employ a consistency test as the method of testing to validate the diagnostic conclusions of the headache CDSS against the gold standard. We hypothesized that there was poor consistency ( $H_{0}$ :kappa = 0) between the new hierarchical fuzzy inference method and the gold standard, and then carried out statistical analysis.

## 2 Methods

### 2.1 Design and construction of a fuzzy diagnostic model based on ICHD-3

#### 2.1.1 Overview

**Figure 1:**
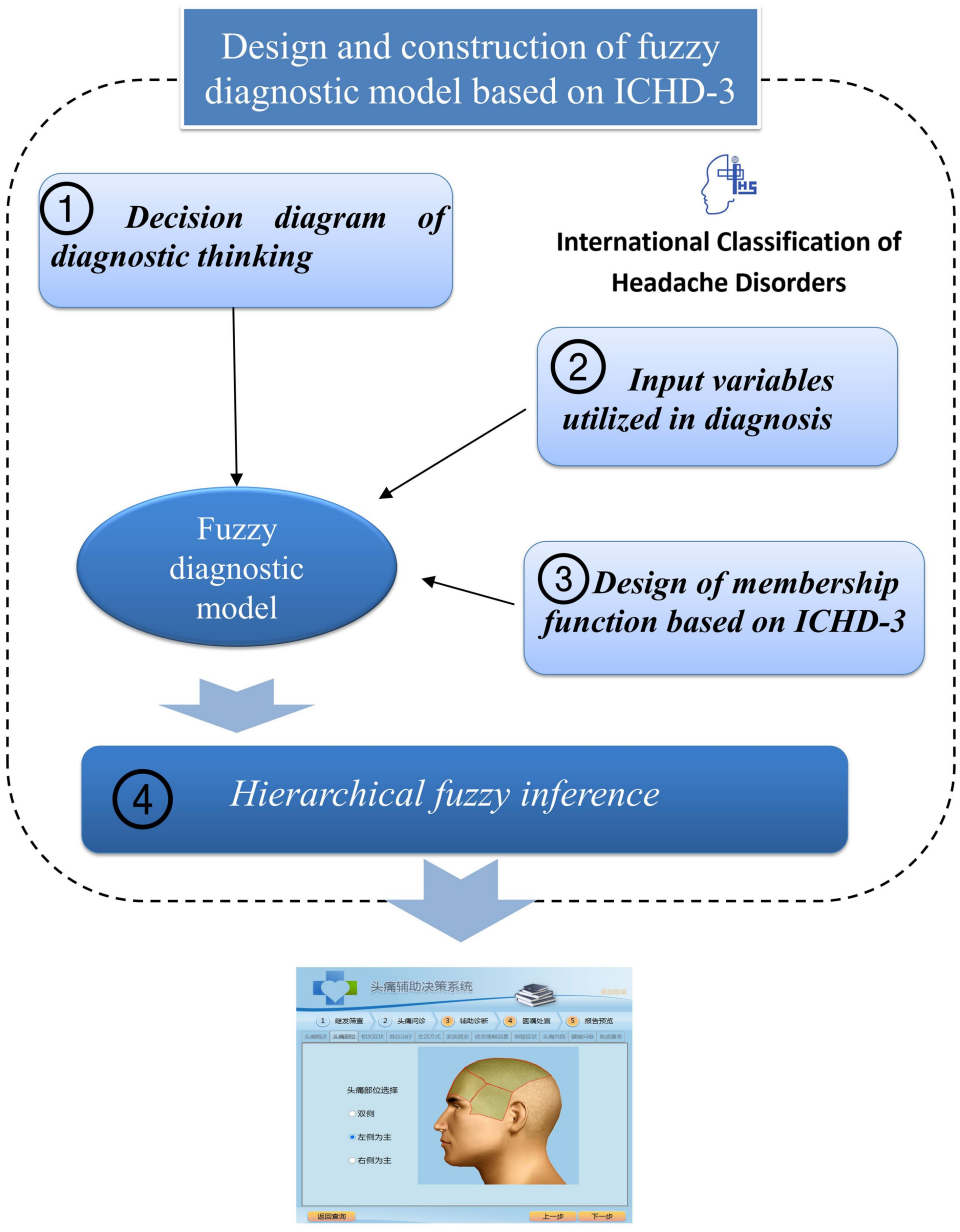
Overview of the fuzzy diagnostic method based on ICHD-3.

As shown in Figure 1, the fuzzy diagnostic method is a four-step process: the first step is the modelling of the diagnostic thinking of headache experts, the second step is determining the fuzzy variables, the third step is the design of the membership functions utilized in fuzzy logic, and the last step is the design of hierarchical fuzzy inference to deal with the fuzzy information.

#### 2.1.2 Modelling of diagnostic thinking

**Figure 2:**
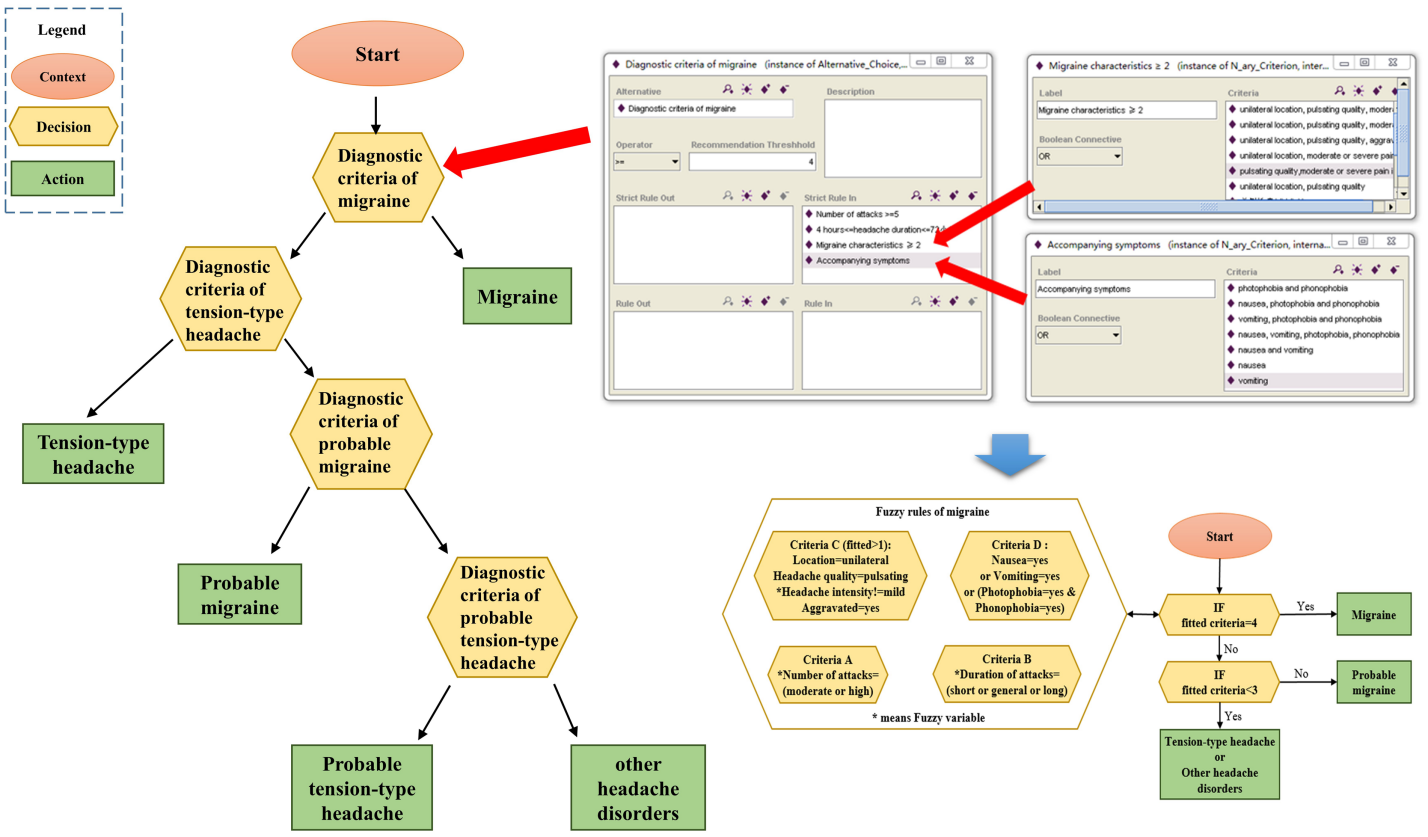
Diagnostic decision diagram model of migraine and TTH.

After excluding secondary headaches, migraine with aura, cluster headaches, and chronic headaches, the headache experts’ diagnostic process for migraine and TTH is summarized as a diagnostic decision diagram, which consists of context nodes, decision nodes and action nodes, as shown in Figure 2. The context node (elliptical node) acts as a starting point of the decision diagram and indicates the beginning of diagnosis. A decision node (hexagon node) means that a decision needs to be made at this point in the process. The red arrow in Figure 2 shows how the ICHD diagnostic criteria are decomposed and ontology expressed in the migraine diagnostic criteria node. Several options are listed in the decision node, and the conditions for choosing an option are listed as well. An action node (rectangular node) is a leaf node of the model, and it needs to be clearly acted on. In this paper, each action node is a diagnosis. With the above components, the decision diagram model of diagnostic thinking on migraine and TTH is built. The decision nodes can be expressed as the corresponding fuzzy rules by fuzzy logic techniques in the subsequent steps.

#### 2.1.3 Input variables utilized in diagnosis

All the patient features mentioned in the diagnostic criteria are included in the diagnostic model. These features can be divided into two categories, numerical features and categorical features, as shown in Table 1. The headache intensity, duration of attacks, and number of attacks are numerical features, whose values need to be classified into fuzzy sets by the predefined membership functions. For example, the number of attacks can be mapped into three fuzzy sets, denoted as {low, moderate, high}, according to the ICHD-3. Other patient features are categorical features, whose sets of possible values are indicated by the ICHD-3 and can be represented by classical two-valued logic as {yes, no}.

**Table 1:** Patient features utilized in the diagnosis of migraine and tension-type headache.

Numerical features	
Name	Fuzzy set
Headache intensity	{mild, moderate, severe}
Duration of attacks	{very short, short, moderate, long, very long}
Number of attacks	{low, moderate, high}

Categorical features	
Name	Crisp set
Headache quality	{pulsating, non-pulsating}
Headache location	{unilateral, bilateral}
Aggravation by or causing avoidance of routine physical activity	{yes, no}
Nausea	{yes, no}
Vomiting	{yes, no}
Photophobia	{yes, no}
Phonophobia	{yes, no}

#### 2.1.4 Design of membership functions based on ICHD-3

**Figure 3:**
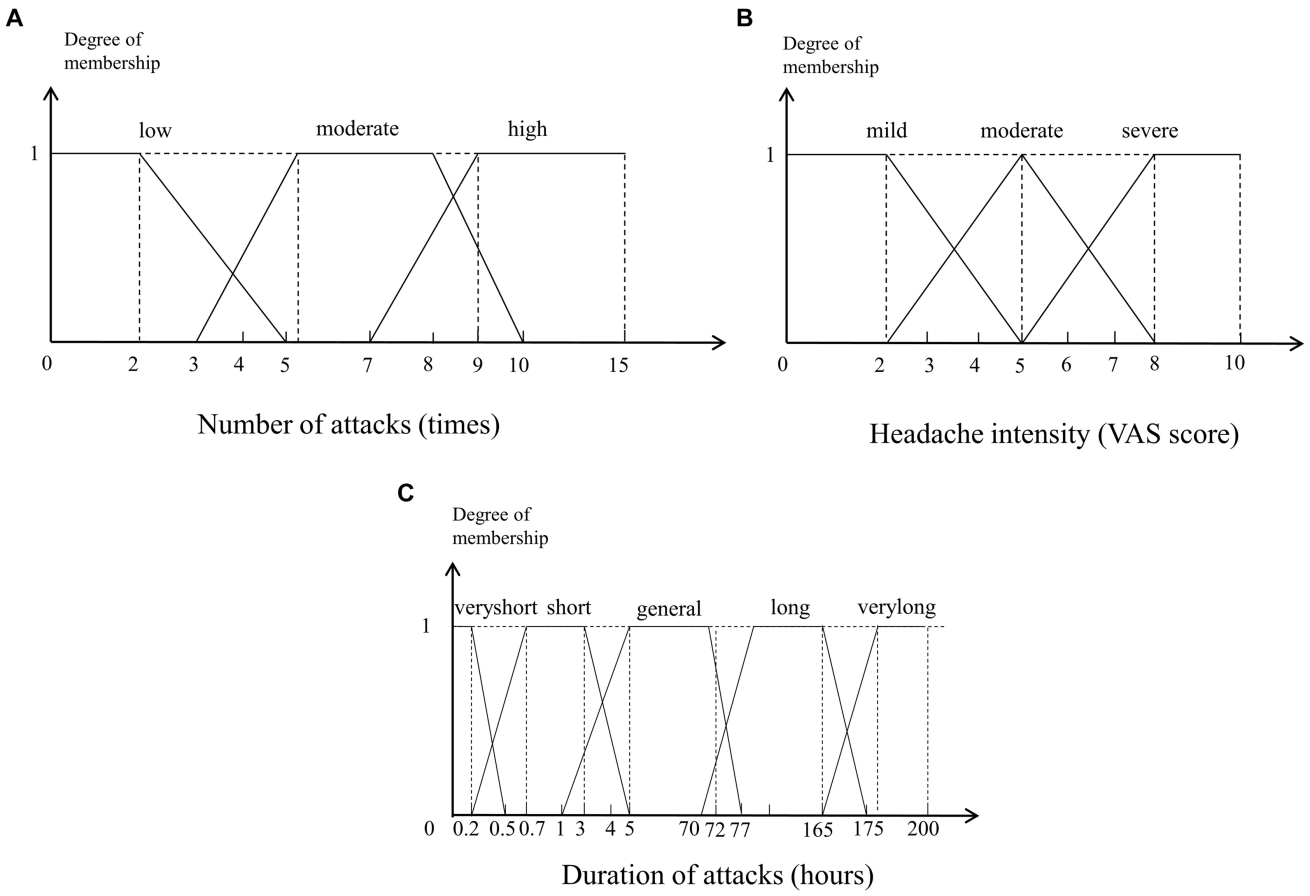
Membership functions of the numerical variables. **(A)** Number of attacks (times). **(B)** Headache intensity (VAS score). **(C)** Duration of attacks (hours).

Membership functions are utilized in the fuzzification and defuzzification steps of a fuzzy logic system to map the non-fuzzy input values to fuzzy linguistic terms and vice versa. The shapes of commonly used membership functions are triangle, trapezoid, rectangle, etc. The membership functions of fuzzy sets are often designed by domain experts based on their experience, so in this paper, we design the membership functions based on ICHD-3. For example, in the diagnostic criteria of migraine, at least five attacks are required, so 5 is the boundary value of the number of attacks. For TTH, the number of attacks should be at least 10, so 10 is also a key boundary value. The membership function of the number of attacks is as shown in Figure 3A. Similarly, for the headache intensity and duration of attacks, we can obtain their membership functions as shown in Figures 3B,C.

#### 2.1.5 Hierarchical fuzzy inference

**Figure 4:**
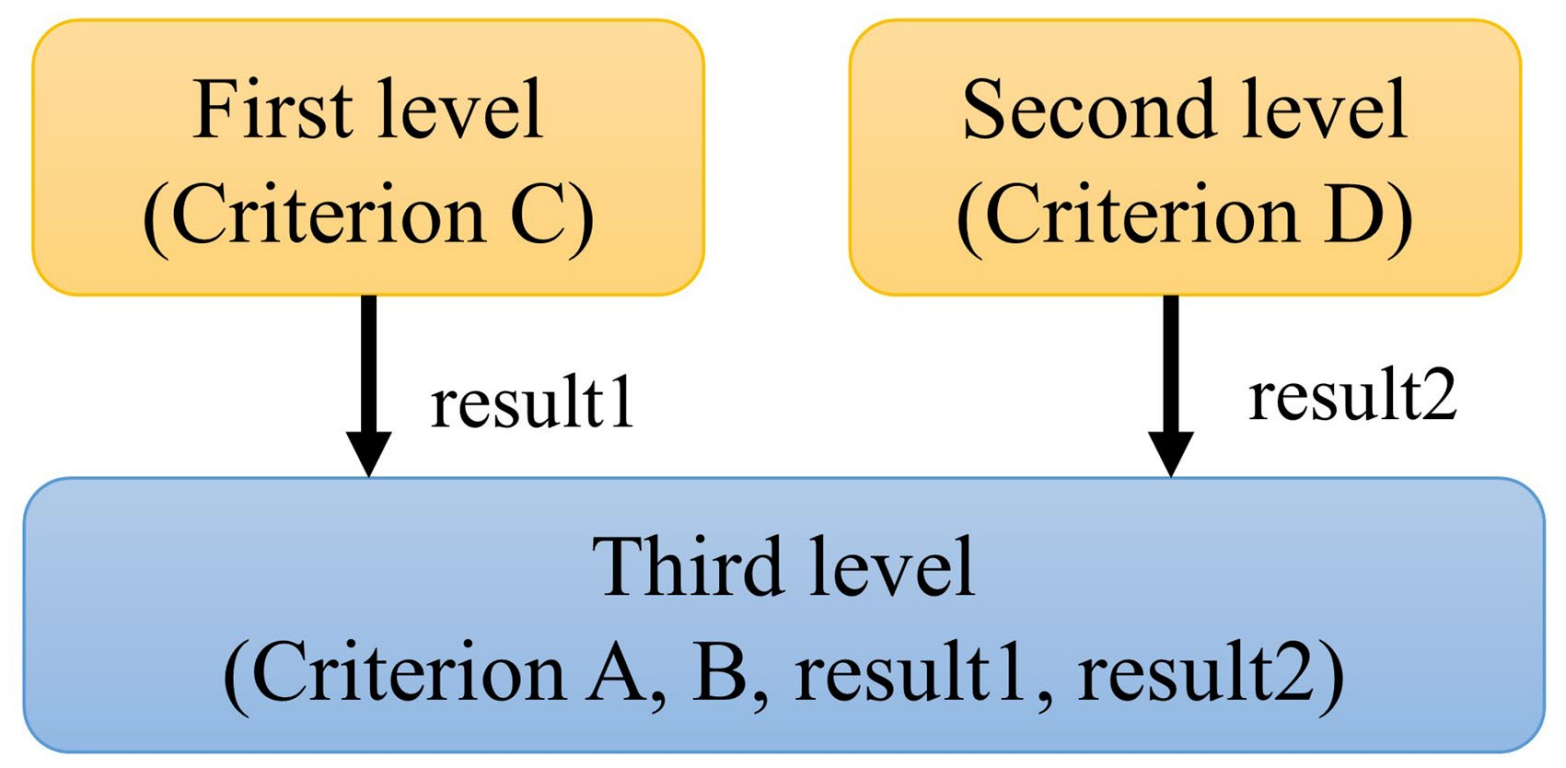
Hierarchical fuzzy inference based on ICHD-3.

To express the diagnostic criteria in ICHD-3 more exactly, we designed a hierarchical fuzzy method with three levels, as shown in Figure 4.

The first level takes criterion C as a small fuzzy system and then adds its inference results into another large fuzzy system. The second level uses traditional two-valued logic to judge whether the patient’s symptoms fulfil criterion D. The third level is the final fuzzy system, which takes the results of the previous two levels as input variables and then carries out a fuzzy inference process. Table 2 shows examples of fuzzy rules, in which fit and unfit indicate whether diagnostic criterion C is fulfilled.

**Table 2:** Examples of fuzzy rules.

#Rule	Rule
1	IF ***headachelocation*** is bilateral and ***headachequality*** is unthrobbing and ***headacheintensity*** is *mild* and ***physicalactivity*** is yes, then result is unfit
2	IF ***headachelocation*** is bilateral and ***headachequality*** is unthrobbing and ***headacheintensity*** is *moderate* and ***physicalactivity*** is yes, then result is fit
3	IF ***numberofattacks*** is *low* and ***headacheduration*** is *general* and criteria C is no and criteria D is yes, then typeofheadache is others
4	IF ***numberofattacks*** is *moderate* and ***headacheduration*** is *general* and criteria C is no and criteria D is yes, then typeofheadache is probablemigraine

The bold font indicates variables in the rules.

Then, in the last step, the weight-based defuzzifier is computed using the results of the membership functions as values and the activation degrees (*α*) as weights. The activation degree of each fuzzy rule can be computed according to the value of the input variables. Taking the first rule in Table 2 as an example, its activation degree is calculated by Equation (1):


(1) $$\begin{array}{c} \alpha =\mu_{\mathrm{unilateral}}\left(x_{\mathrm{headachelocation}}\right)\wedge \mu_{\mathrm{throbbing}}\left(x_{\mathrm{headachequality}}\right)\\ \wedge \mu_{\mathrm{mild}}\left(x_{\mathrm{headacheintensity}}\right) \end{array}$$

where $\mu_{\mathrm{mild}}\left(x_{\mathrm{headacheintensity}}\right)$ is the membership degree of $x_{\mathrm{headacheintensity}}$ in the mild fuzzy set.

Considering that one of the output variables Typeofheadache (for migraine) contains the constant terms *migraine* = 2, *probablemigraine* = 1, and *others* = 0, its crisp output value computed with the weighted-average defuzzifier is given by:


(2) $$f_{\mathrm{Typeofheadache}}\left(\begin{array}{l} x_{\mathrm{Numberofattack}},\;\\ x_{\mathrm{Headacheduration}},\;\\ x_{\mathrm{criterionC}},\;x_{\mathrm{criterionD}} \end{array}\right)=\frac{\begin{array}{l} \alpha_{1}\mathrm{migraine}+\\ \alpha_{2}\mathrm{probablemigraine}+\\ \alpha_{3}\mathrm{others}\; \end{array}}{\alpha_{1}+\alpha_{2}+\alpha_{3}}$$

The closer the output value is to each given constant term, the more likely the CDSS is to diagnose the patient as suffering from that type of headache. For example, the closer the output value is to 2, the more likely it is that the patient is suffering from migraine. The closer the output value is to 0, the more likely the patient is to have another type of headache. Similarly, the output variable Typeofheadache (for tension-type headache) is computed by:


(3) $$f_{\mathrm{Typeofheadache}}\left(\begin{array}{l} x_{\mathrm{Numberofattack}},\;\\ x_{\mathrm{Headacheduration}},\;\\ x_{\mathrm{criteriaC}},x_{\mathrm{criteriaD}} \end{array}\right)=\frac{\begin{array}{l} \alpha_{1}\mathrm{Tension type headache}+\\ \alpha_{2}\mathrm{Probable tension type headache}+\\ \alpha_{3}\mathrm{others} \end{array}}{\alpha_{1}+\alpha_{2}+\alpha_{3}}$$

where *tensiontypeheadache* = 2, *probabletensiontypeheadache* = 1, and *others* = 0.

### 2.2 Participants and validation

#### 2.2.1 Validation

**Figure 5:**
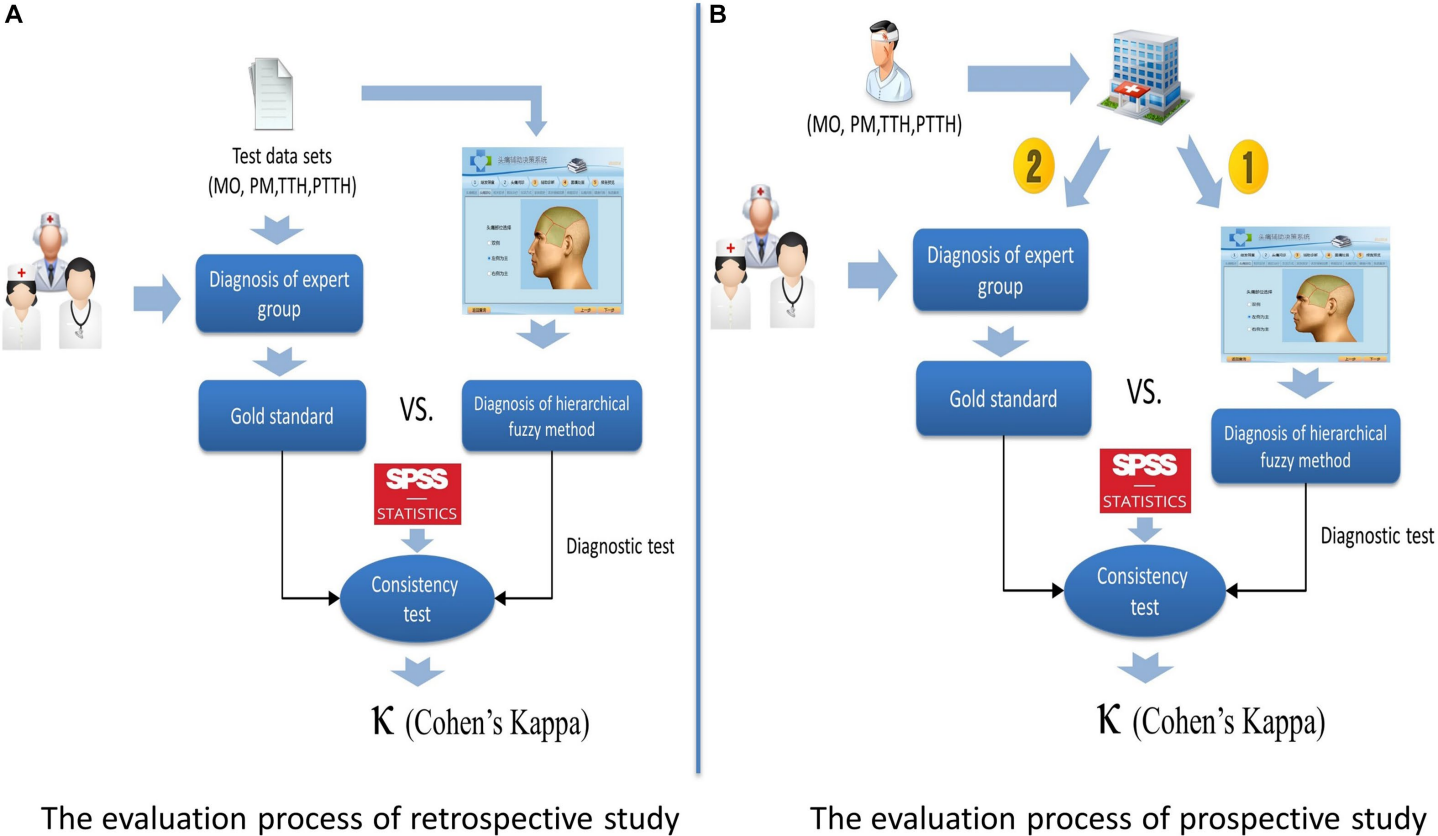
Evaluation method of the fuzzy-based CDSS. **(A)** The evaluation method of the fuzzy-based CDSS. **(B)** The evaluation process of prospective study.

To evaluate the validity of the presented method, a retrospective study and a prospective study were designed and conducted in Chinese PLA General Hospital respectively. For the retrospective study, the process of evaluation was as follows: first, the diagnosis in each case was reconfirmed by three headache experts and the diagnosis of the expert group was regarded as the gold standard; then, the fuzzy-based CDSS made diagnosis according to the clinical symptoms of each case input by a neurologist who did not know the experts’ diagnosis. For the prospective study, the fuzzy-based CDSS was applied in a real clinical environment. The process of evaluation was as follows: when a patient came to the headache clinic, his/her clinical symptoms were input into the CDSS after an in-depth clinical interview was conducted with a neurologist, and a diagnosis conclusion was made by the CDSS; then the patient would enter into the three headache experts’ offices, and independent diagnoses was made by the three experts respectively. The final decision of the expert group was by a simple majority. The diagnostic procedures of the headache expert group and the fuzzy-based CDSS were independent and completed on the same day. The process of retrospective study and prospective study are shown in Figure 5.

#### 2.2.2 Participants

The retrospective study included the electronic medical records of 343 consecutive migraine and TTH patients enrolled at the headache clinic of the International Headache Center at Chinese PLA General Hospital between November 2018 and February 2019. Eighteen patients were excluded from the analysis because of missing data. Two patients diagnosed with migraine with aura were also excluded because it is very easy to be diagnosed by our previous rule-based methods due to the specificity of aura and is not within the scope of our study. The diagnoses of the remaining cases (325 cases) were migraine without aura (MO) (131, 40.31%), PM (80, 24.62%), infrequent/frequent TTH (70, 21.54%), and PTTH (44, 13.54%). The prospective study was also conducted in the headache clinic of Chinese PLA General Hospital from June 2022 to January 2023. Three hundred and eighty four patients were enrolled and 4 patients were excluded because of migraine with aura. The diagnoses of remaining cases (380 cases) were MO (179, 47.11%), PM (62, 16.32%), infrequent/frequent TTH (115, 30.26%), and PTTH (24, 6.31%). The demographics and clinical characteristics of the retrospective study cohort and the prospective study cohort are shown in Table 3.

**Table 3:** Basic demographic and clinical characteristics of the study cohort and the prospective study cohort.

Characteristics	Retrospective study (*N* = 325)	Prospective study (*N* = 380)
Sex (male/female)	109 (33.5%)/216 (66.5%)	86 (22.6%)/294 (77.4%)
Age (mean, SD)	38.7 (12.7)	39.6 (12.3)
Duration of attacks (mean, SD) (hours)	17.0 (28.2)	24.3 (27.1)
Course of headache (mean, SD) (months)	75.4 (94.3)	91.2 (103.5)
Number of attacks (<5/6–9/>10)	22 (6.8%)/21 (6.4%)/282 (86.8%)	18 (4.7%)/34 (8.9%)/328 (86.3%)
Location (unilateral/bilateral)	91 (28%)/234 (72%)	178 (46.8%)/202 (53.2%)
Headache quality (pulsating/non-pulsating/other)	172 (52.9%)/142 (43.7%) /11 (3.4%)	230 (60.5%)/137 (36.1%)/13 (3.4%)
Headache intensity (mean, SD) (VAS score)	6.0 (1.8)	6.4 (1.5)
Aggravated by routine physical activity (yes/no)	151 (46.5%)/174 (53.5%)	225 (59.2%)/155 (40.8%)
Nausea (%)	33.8	47.1
Vomiting (%)	16.9	50.8
Photophobia (%)	26.8	48.9
Phonophobia (%)	31.1	62.6

#### 2.2.3 Statistical analysis

A diagnostic test is a procedure performed to confirm or determine the presence of disease in an individual suspected of having a disease. In this study, the diagnostic tests were performed by comparing the diagnosis of fuzzy-based CDSS and the headache experts’ decisions for each headache case both in the retrospective study and the prospective study. SPSS for Windows (Version 16.0) was used for the statistical analysis. We measured the diagnostic performance of the proposed method using the following metrics: sensitivity, specificity, PPV, NPV, total consistency rate (*π*) and Youden index.

Sensitivity refers to the proportion of those who have the disease (when judged by the “Gold Standard”) that received a positive result on this test. Specificity refers to the proportion of those who do not have the disease (when judged by the “Gold Standard”) that received a negative result on this test. The total consistency rate ( π ) represents the degree to which the diagnostic results of the diagnostic method to be evaluated accord with the results of the gold standard diagnostic method. The Youden index indicates the ability of diagnostic methods to detect patients and nonpatients. The mistaken diagnosis rate ( α ), also known as the false positive rate, is the probability that a healthy person is diagnosed as a patient by the diagnostic method to be evaluated. The omission diagnostic rate ( β ), also known as the false negative rate, represents the probability that a patient is evaluated as healthy by the diagnostic method to be evaluated. The positive predictive value (PPV) denotes the probability that subjects with a positive diagnosis truly have the disease. The negative predictive value (NPV) is the probability that subjects with a negative diagnosis truly do not have the disease.

In addition, a consistency test was also performed, and Cohen’s kappa (*κ*) was calculated for the agreement between diagnoses. The consistency test cannot only indicate whether two methods are consistent but also evaluate the degree of consistency by calculating Cohen’s kappa value. Kappa is calculated by Equation (4):


(4) $$\mathrm{Kappa}=\frac{P_{A}-P_{e}}{1-P_{e}}$$

where $P_{A}$ is the actual observed consistency rate and $P_{e}$ is the expected consistency rate. Generally, if *κ* ≥ 0.85, the consistency is considered very good; if 0.6 ≤ *κ* < 0.85, the consistency is good; if 0.45 ≤ *κ* < 0.6, the consistency is moderate; and if *κ* < 0.45, the consistency is poor. A 5% level of significance and 95% confidence intervals (CI) were utilized in this study.

#### 2.2.4 Real case study

Finally, we selected two real cases from the CDSS headache database and performed case studies by demonstrating the computational procedure of our proposed method to show the feasibility of the method.

## 3 Results

### 3.1 The GUI of the fuzzy-based headache CDSS

**Figure 6:**
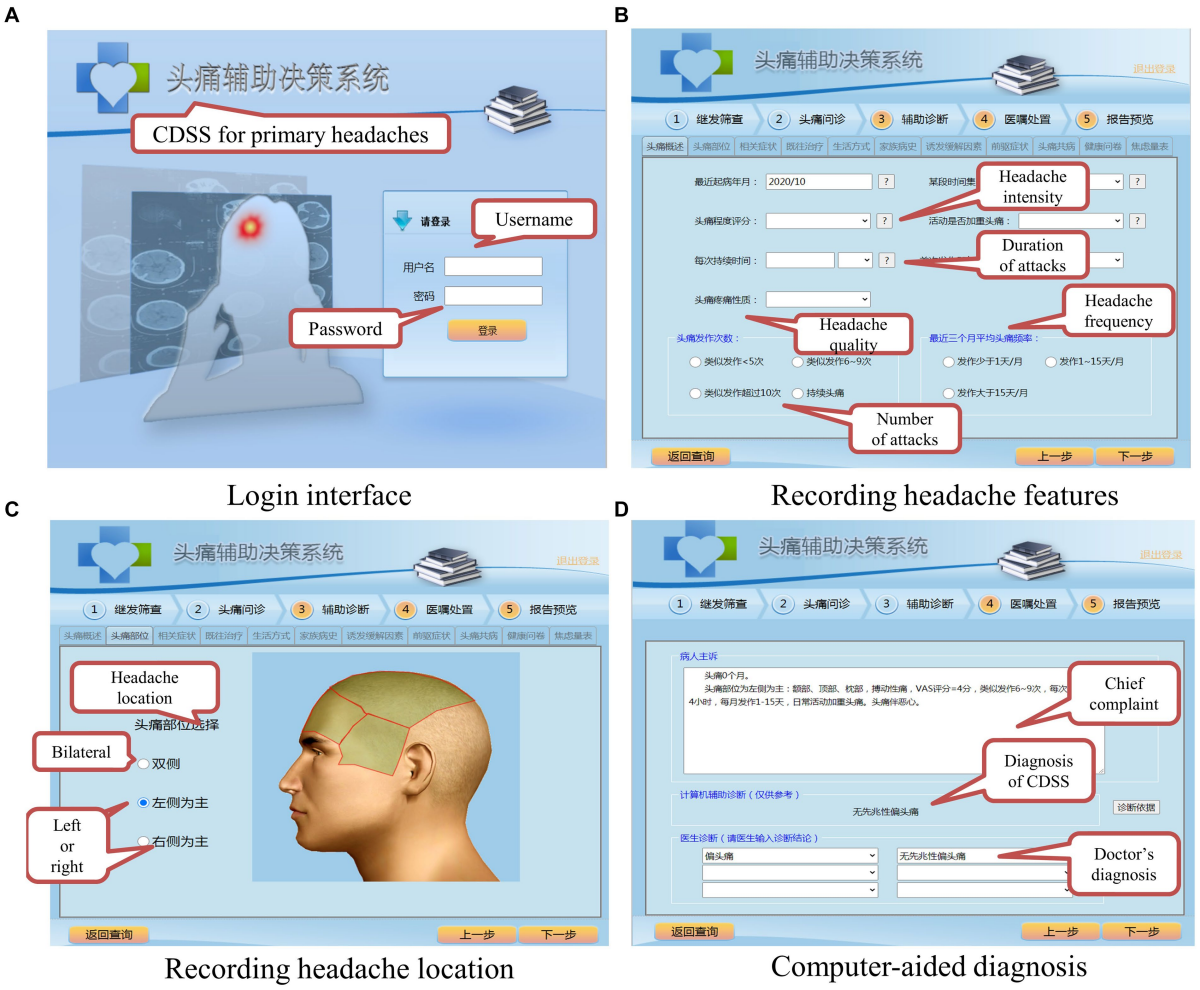
Main functions of the fuzzy-based CDSS. **(A)** Login interface. **(B)** Recording headache features. **(C)** Recording headache location. **(D)** Computer-aided diagnosis.

The main functions of the CDSS in this paper are shown in Figure 6. The CDSS is available at the website and can be visited with a web browser on desktop computers and mobile devices. Figure 6A shows the login interface; every doctor authorized to use the system is assigned a username and a password. Figure 6B shows the user interface for recording the patient’s headache features. All the input items of the system interface come from the ICHD-3, and they include all the necessary information for diagnosis. The doctor can interview a patient according to the tips in the GUI, which can help to save much time and prevent the doctor from missing critical information. Figure 6C shows the user interface for recording the headache location. After the previous step, the CDSS can make a diagnosis by fuzzy logic, as shown in Figure 6D. The doctor needs to decide whether to accept the diagnosis of the CDSS. If not, the doctor needs to input a different diagnosis into the CDSS. The doctor can click on the “Diagnostic criteria” button if it is not clear why the CDSS has made a certain diagnosis, and the CDSS will display the latest diagnostic criteria to help the doctor better understand the ICHD-3.

### 3.2 Diagnostic performance of the fuzzy-based CDSS

#### 3.2.1 Retrospective study

As shown in Table 4, the fuzzy-based CDSS correctly recognized 128/131 patients (97.71%) with migraine without aura (MO), 69/70 patients (98.57%) with TTH, 73/80 patients (91.25%) with probable migraine (PM), and 40/44 patients (90.91%) with probable tension-type headache (PTTH).

**Table 4:** Agreement between the fuzzy-based CDSS and headache expert group diagnoses.

Fuzzy-based CDSS	Headache expert group				
	MO	TTH	PM	PTTH	Total
MO	128	0	0	0	128
TTH	0	69	0	0	69
PM	3	0	73	0	76
PTTH	0	1	0	40	41
Others	0	0	7	4	11
Total	131	70	80	44	325

MO, migraine without aura; TTH, tension-type headache; PM, probable migraine; PTTH, probable tension-type headache; others, other headache disorders.

To demonstrate the feasibility of the proposed method, we also compared the diagnosis of the ICHD-rule-based CDSS with the gold standard. As shown in Tables 4, 5, compared with the ICHD-rule-based CDSS, the fuzzy-based CDSS shows significantly improved diagnostic classification performance (95.4% (310/325) vs. 90.2% (293/325), *p* < 0.01).

**Table 5:** Agreement between the ICHD-rule-based CDSS and the headache expert group diagnoses.

ICHD-rule-based CDSS	Headache expert group				
	MO	TTH	PM	PTTH	Total
MO	126	0	0	0	126
TTH	0	64	0	0	64
PM	5	3	69	0	77
PTTH	0	2	0	34	36
Others	0	1	11	10	22
Total	131	70	80	44	325

MO, migraine without aura; TTH, tension-type headache; PM, probable migraine; PTTH, probable tension-type headache; others, other headache disorders.

The sensitivity, specificity, total consistency rate (*π*), positive predictive value (PPV), negative predictive value (NPV) and Youden index of both CDSSs are shown in Table 6. The diagnostic ability of the two methods is similar in the diagnosis of MO, but compared with the ICHD-rule-based method, the classification performance of the fuzzy-based method is greatly improved in the diagnosis of TTH, PTTH and PM. In particular, for PM and PTTH with less typical symptoms, the fuzzy-based method significantly improves the Youden index by the fuzzy processing of the numerical boundaries of headache features, which shows that it can effectively reduce the mistake diagnostic rate and omission diagnostic rate. In general, the hierarchical fuzzy-based CDSS is better than the ICHD-rule-based CDSS in the diagnosis of PM and PTTH.

**Table 6:** Comparison between the rule-based CDSS and fuzzy-based CDSS for diagnostic tests.

	ICHD-rule-based CDSS				Fuzzy-based CDSS			
	MO	PM	TTH	PTTH	MO	PM	TTH	PTTH
Sensitivity(%) (95% CI)	96.18 90.87-98.59	86.25 76.31-92.61	91.43 81.65-96.47	77.27 61.78-88.01	97.71 92.94-99.41	91.25 82.25-96.11	98.57 91.23-99.93	90.91 77.42-97.05
Specificity(%) (95% CI)	100 97.58-100	96.73 93.43-98.47	100 98.15-100	99.29 97.17-99.88	100 97.58-100	98.78 96.17-99.68	100 98.15-100	99.64 97.72-99.98
PPV(%) (95% CI)	100 96.31-100	89.61 80.03-95.09	100 92.95-100	94.44 79.99-99.03	100 96.37-100	96.05 88.12-98.98	100 93.43-100	97.56 85.59-99.87
NPV(%) (95% CI)	97.49 93.91-99.07	95.56 91.98-97.65	97.70 94.82-99.06	96.54 93.53-98.23	98.48 95.25-99.61	97.19 94.05-98.76	99.61 97.50-99.98	98.59 96.19-99.55
Youden index	0.9618	0.8298	0.9143	0.7656	0.9771	0.9003	0.9857	0.9055
**π**	0.9846	0.9415	0.9815	0.9631	0.9908	0.9692	0.9969	0.9846

MO: migraine without aura; TTH: tension-type headache; PM: probable migraine; PTTH: probable tension-type headache; others: other headache disorders.

#### 3.2.2 Prospective study

In the prospective study, the fuzzy-based CDSS correctly recognized 164/179 (91.62%) of MO, 106/115 (92.17%) of TTH, 53/62 (85.48%) of PM, and 21/24 (87.50%) of PTTH. The agreement between the fuzzy-based CDSS and the gold standard is shown in Table 7.

**Table 7:** Agreement between the fuzzy-based CDSS and the gold standard in the prospective study.

Fuzzy-based CDSS	Headache expert group				
	MO	TTH	PM	PTTH	Total
MO	164	4	3	0	171
TTH	5	106	5	2	118
PM	5	0	53	1	59
PTTH	1	3	1	21	26
Others	4	2	0	0	6
Total	179	115	62	24	380

MO, migraine without aura; TTH, tension-type headache; PM, probable migraine; PTTH, probable tension-type headache; others, other headache disorders.

The main indicators of diagnostic test for the fuzzy-based CDSS are shown in Table 8. From Table 8, we can see that although the diagnostic sensitivity of each headache decrease slightly compared with that of the retrospective study, the total consistency rate (*π*) remains at a high level (>90%). This means that our fuzzy-based method has good stability even in the real clinical environment.

**Table 8:** The diagnostic performance of the fuzzy-based CDSS in the prospective study.

	MO	PM	TTH	PTTH
Sensitivity(%) (95% CI)	91.62 86.30-95.07	85.48 73.72-92.75	92.17 85.26-96.13	87.50 66.54-96.71
Specificity(%) (95% CI)	96.52 92.66-98.47	98.11 95.74-99.23	95.47 92.02-97.53	98.60 96.56-99.48
PPV(%) (95% CI)	95.91 91.42-98.19	89.83 78.50-95.80	89.83 82.56-94.40	80.77 60.02-92.69
NPV(%) (95% CI)	92.82 88.21-95.79	97.20 94.56-98.63	96.56 93.36-98.31	99.15 97.33-99.78
Youden index	0.8814	0.8359	0.8764	0.8610
**π**	0.9421	0.9605	0.9447	0.9789

MO: migraine without aura; TTH: tension-type headache; PM: probable migraine; PTTH: probable tension-type headache.

### 3.3 Consistency test of the fuzzy-based CDSS

We performed a consistency test between the fuzzy-based method and the gold standard to evaluate the consistency of the two methods. The results of the consistency test are shown in Table 9. In the retrospective study, the values of *κ* for MO (0.984 ± 0.018, *p* < 0.001), TTH (0.991 ± 0.018, *p* < 0.001), PM (0.916 ± 0.051, *p* < 0.001), PTTH (0.932 ± 0.059, *p* < 0.001) are all much greater than 0.85, and in the prospective study, the values of *κ* for MO (0.884 ± 0.047, *p* < 0.001), TTH (0.870 ± 0.055, *p* < 0.001), PM (0.853 ± 0.073, *p* < 0.001), PTTH (0.827 ± 0.118, *p* < 0.001) are all greater than 0.80, so we reject the null hypothesis ( $H_{0}$ : kappa = 0) proposed in the Introduction section. These results indicate that there is good consistency between the fuzzy-based CDSS and the headache experts, and they show that the diagnostic ability of the fuzzy-based method is very close to that of headache experts.

**Table 9:** Consistency test and Cohen’s kappa for each headache between the gold standard and the fuzzy-based CDSS in the retrospective study and prospective study.

	Cohen’s kappa (retrospective study)	Cohen’s kappa (prospective study)
MO	0.984 ± 0.018, *p* < 0.001	0.884 ± 0.047, *p* < 0.001
TTH	0.991 ± 0.018, *p* < 0.001	0.870 ± 0.055, *p* < 0.001
PM	0.916 ± 0.051, *p* < 0.001	0.853 ± 0.073, *p* < 0.001
PTTH	0.932 ± 0.059, *p* < 0.001	0.827 ± 0.118, *p* < 0.001

MO, migraine without aura; TTH, tension-type headache; PM, probable migraine; PTTH, probable tension-type headache.

### 3.4 Case study of the fuzzy-based CDSS

To further illustrate the effectiveness of the proposed hierarchical fuzzy method, we selected two cases from the database as case studies.

#### 3.4.1 Case 1

A 42-year-old woman visited our hospital with a complaint of 4 headache episodes over the last 3 months, and her headache had the following features: a unilateral location, a pulsating quality, mild to moderate pain, and aggravation by routine physical activity. Each episode lasted from 2 h to half a day. The pain was accompanied by symptoms of nausea and vomiting, with no photophobia or phonophobia. CT scanning was performed on December 1, 2018, and the results were normal. Her father has similar headaches.

It is notable that the patient’s number of attacks was 4, and this did not fulfil the minimum diagnostic criteria for migraine (at least 5 attacks). Additionally, criterion B for migraine was not met. In theory, the patient’s symptoms did not fulfil any diagnostic criteria in ICHD-3. However, clearly, the patient had a migraine-like attack, so the diagnosis of the headache experts was probable migraine.

In the fuzzy-based CDSS, the membership degree of 4 for the “low” category is 1/3 and that for the “moderate” category is 1/2. In Equation 2, $\alpha_{1}$ = 0.25, $\alpha_{2}$ = 0.75, $\alpha_{3}$ = 0.3333, and $f_{\mathrm{Typeofheadache}}$ for migraine is 0.9375, which is closer to the probable migraine category (*migraine* = 2, *probablemigraine* = 1, and *others* = 0). In Equation 3, $\alpha_{1}$ = 0, $\alpha_{2}$ = 0, $\alpha_{3}$ = 1.33333, and $f_{\mathrm{Typeofheadache}}$ for tension-type headache is 0, which comes closer to the other headache category (*tensiontypeheadache* = 2, *probabletensiontypeheadache* = 1, and *others* = 0). Taking into account these two results, the patient was diagnosed with probable migraine by the fuzzy-based CDSS.

#### 3.4.2 Case 2

A 38-year-old woman reported that the number of attacks was 3 in the past 3 months, and each attack lasted almost 20 min. Her headache features were bilateral location, no pulsating quality, VAS score of 3, and no aggravation by routine physical activity. The pain was not accompanied by symptoms of nausea, vomiting, photophobia or phonophobia. No abnormality was found on routine examinations.

Although criterion A (number of attacks) and criterion B (duration of attacks) were not fulfilled according to the diagnostic criteria of tension-type headache, the patient was still diagnosed with probable tension-type headache-like attacks by the expert group because of the lack of accompanying symptoms and fully fulfilling criterion C of tension-type headache. In Equation 2, $\alpha_{1}$ = 0, $\alpha_{2}$ = 0, $\alpha_{3}$ = 1.4, $f_{\mathrm{Typeofheadache}}$ = 0. In Equation 3, $\alpha_{1}$ = 0, $\alpha_{2}$ = 0.7333, $\alpha_{3}$ = 0.6666, and $f_{\mathrm{Typeofheadache}}$ = 0.5238, which is closer to 1, so synthesizing these two results, the diagnosis of the fuzzy-based CDSS was probable tension-type headache, which is the same as that of the expert group.

## 4 Discussion

To solve the problem of the imprecise description of fuzzy headache features (i.e., the duration of attacks, number of attacks and headache intensity) caused by recall bias and subjective bias, we proposed a hierarchical fuzzy headache diagnostic inference method based on ICHD-3. This hierarchical method comprises two levels. First, we match patients’ symptoms against the diagnostic criteria C and D for migraine and tension-type headache, as outlined in the ICHD-3. Second, the outcomes from these matches are integrated with the already fuzzified criteria A and B for the second-step diagnosis. This hierarchical method serves as a complement to the rule-based reasoning method and is activated only when a probable migraine or probable TTH diagnosed by the rule-based reasoning. Furthermore, a fuzzy-based CDSS was established, and the validity of the CDSS was evaluated by a retrospective study and a prospective study. The evaluation results show that the fuzzy-based CDSS has good diagnostic performance and that its diagnosis results have good consistency with headache experts’ diagnoses. We hope that the developed headache CDSS can help non specialists distinguish between probable migraine and probable TTH in primary hospitals.

### 4.1 Comparison to prior works

Currently, some scholars have also tried to develop various headache CDSSs based on different artificial intelligence methods. Generally, there are two feasible methods. One is based on the diagnostic criteria in the ICHD, which can be regarded as a knowledge-driven method. Some scholars have established heuristic rules based on headache diagnosis (17, 18). The other type of method is based on clinical data. With the development of data science, some scholars have tried to build intelligent computer-aided diagnosis models based on machine learning techniques (19–23), which could be called data-driven methods. Each of these methods has merits and shortcomings, but considering the acceptability to clinicians, CDSSs based on ICHD-3 may be a better option. This is because it is difficult to explain the mathematical principles of the models generated by machine learning algorithms to doctors, and these models are similar to “black boxes” for doctors. However, traditional rule-based reasoning is unable to handle the fuzzy boundaries of headache features, and few scholars have paid attention to this problem. Therefore, in this study, we proposed a “rule-based reasoning + hierarchical fuzzy logic” method, a new hybrid intelligent technique, to develop a fuzzy-based headache CDSS. Rule-based reasoning is the “backbone,” which is used to express the logic of the reasoning, and fuzzy logic is utilized to deal with the fuzzy boundary values of some features, such as the headache intensity, number of attacks, and duration of attacks. With the ability to imitate experts dealing with boundary values, fuzzy logic enhances the capability of the CDSS to handle uncertain information. Hence, these two intelligent methods used in the CDSS combine the advantages of both and are complementary to each other. Compared with the routine CDSS method, all the performance metrics of the fuzzy-based CDSS are significantly improved to varying degrees. Moreover, this work shows the potential to be extended to other primary headaches as well.

The fuzzy-based headache CDSS is designed for doctors who are not familiar with the diagnostic criteria of primary headaches and can help general practitioners and junior doctors diagnose headaches in a clinical setting, which is meaningful for headache diagnosis at the rural and community levels. It is hoped that this will change the status quo of the low diagnosis rate by general practitioners in China at present. In addition, the web-based CDSS is convenient for doctors to use to access the latest diagnostic criteria of primary headaches.

### 4.2 Limitation

According to the results in Table 6, although our method is better than the routine CDSS, there is still a certain gap between our method and the gold standard. After in-depth analysis, we think that the main reason for the difference is the completeness of diagnostic model. For example, if two of the patient’s symptoms do not fulfill the diagnostic criteria slightly, our method cannot draw a definite diagnosis, while doctors can diagnose her as probable migraine through other relevant symptoms, such as the menstrual cycle.

There are also some other limitations of this study. First, this study focused only on migraine and TTH and did not cover other primary headache disorders, so we will design a similar method to address cluster headaches and other primary headache disorders in the next stage. Second, the inference process of the fuzzy-based CDSS does not include the weight of each headache feature. Many studies have shown that adding different weights to each attribute is helpful in improving the diagnostic accuracy of a CDSS. In addition to the weights of the headache features, we will add a weight to each fuzzy rule to make the inference conclusion more accurate. Last, but importantly, the amount of data we currently used for the retrospective and prospective study in this paper is quite limited. In the future, as more and more data accumulates, we will also conduct large-scale, multi center clinical validation of the system to ensure the reliability of our conclusions.

## 5 Conclusion

In this paper, a hierarchical fuzzy inference method was designed, and a fuzzy-based headache CDSS was developed, which solved the problem of fuzzy headache features that are caused by recall bias and subjective bias. The evaluation results proved that the hierarchical fuzzy method can diagnose migraine and tension-type headache with high sensitivity and specificity, better than the routine CDSS method, and its diagnostic level is close to that of headache experts. In the future, we aspire to integrate the latest artificial intelligence technologies, encompassing fuzzy knowledge graph and fuzzy deep learning, into the realm of headache CDSS, with the goal of enhancing not only the diagnostic accuracy but also the interpretability of these systems, ultimately facilitating improved headache diagnosis accuracy among general practitioners, junior doctors, and community doctors.

## Data Availability

The raw data supporting the conclusions of this article will be made available by the authors, without undue reservation.
